# Poly(lactic-co-glycolic acid) nanoparticles and microparticles for peptide delivery: release mechanisms and controlling factors

**DOI:** 10.5599/admet.3091

**Published:** 2025-12-10

**Authors:** Mohammadmahdi Eshaghi, Marzieh Dehghani, Azam Abedi, Mehrdad Moosazadeh Moghaddam, Ramezan Ali Taheri

**Affiliations:** 1 Nanobiotechnology Research Center, New Health Technologies Institute, Baqiyatallah University of Medical Sciences, Tehran, Iran; 2 Department of Biophysics, College of Biological Sciences, Tarbiat Modares University, Tehran, Iran; 3 Tissue Engineering and Regenerative Medicine Research Center, New Health Technologies Institute, Baqiyatallah University of Medical Sciences, Tehran, Iran

**Keywords:** Poly(lactic-co-glycolic acid), drug delivery, particle, nanomaterial

## Abstract

**Background and purpose:**

Therapeutic peptides offer high potency but are limited by rapid degradation, poor bioavailability, and the need for frequent dosing. Poly(lactic-co-glycolic acid) (PLGA) nanoparticles and microparticles are effective carriers that protect peptides and enable controlled release. This review summarizes the mechanisms governing peptide release from PLGA particles and identifies formulation factors critical for optimizing therapeutic performance.

**Approach:**

A comprehensive analysis of polymer characteristics, particle design parameters, peptide physicochemical properties, and formulation strategies was conducted using data from recent studies. Release mechanisms, including diffusion, polymer degradation, and erosion, were examined alongside manufacturing methods. The review also evaluates clinical PLGA-based peptide products to highlight translational relevance.

**Key results:**

Peptide release profiles are strongly influenced by PLGA molecular weight, lactide:glycolide ratio, particle size, end-group chemistry, drug loading, and excipients. Lower polymer molecular weight, higher glycolide content, and smaller particle dimensions accelerate release, whereas cationic peptides experience electrostatic retention within degrading matrices. Additives such as PEG, magnesium salts, and chitosan coatings effectively modulate burst release and stability. PLGA systems typically display triphasic release profiles governed by diffusion and erosion. Several PLGA microparticle-based peptide depots have achieved clinical success, although no nanoparticle-based products have yet reached the market due to manufacturing and regulatory challenges.

**Conclusion:**

PLGA nano- and microparticles provide versatile, tunable platforms for sustained peptide delivery. Understanding the interplay between polymer properties, particle architecture, and peptide characteristics is essential for designing next-generation long-acting formulations with improved efficacy and clinical translation.

## Introduction

Recent studies have shown that conventional drug delivery methods face significant limitations in precisely controlling the timing and location of drug release. After administration, the active compound often distributes throughout the body *via* the bloodstream, leading to significant fluctuations in drug concentration. In many cases, plasma levels peak shortly after administration but rapidly decline below the therapeutic threshold. This necessitates frequent and prolonged dosing to maintain effective treatment. To address these challenges, advanced drug delivery systems (DDS) have been developed. These systems are designed to release drugs slowly and continuously or maintain a steady release rate, ultimately enabling targeted delivery to specific tissues or organs [1,2]. Today, polymeric DDSs are emerging as a promising solution to overcome the challenges in drug delivery. Polymeric DDSs have emerged as a versatile and widely explored platform for drug delivery due to their tuneable physicochemical properties, biocompatibility, and ability to protect therapeutic agents from degradation.

These carriers are typically composed of biodegradable synthetic or natural polymers, such as poly(lactic-co-glycolic acid) (PLGA), polyethylene glycol (PEG), chitosan, alginate, or polycaprolactone (PCL). Common types include polymeric nanoparticles and microparticles, micelles, dendrimers, nanogels, and polymersomes, each offering unique structural and functional advantages. For example, micelles enhance the solubility of hydrophobic drugs, dendrimers provide multivalent attachment sites, and nanogels facilitate stimulus-responsive drug release. Additionally, these systems can be functionalized for targeted delivery by conjugating ligands, antibodies, or aptamers to their surface. The advantages of using polymeric DDS are substantial. They can improve the pharmacokinetics and bioavailability of poorly soluble drugs, prolong systemic circulation, and enable controlled or sustained release profiles [3].

These properties reduce dosing frequency and systemic side effects by concentrating the therapeutic effect at the disease site. Furthermore, the biodegradability of polymers such as PLGA, PCL, *etc.* ensures safe clearance from the body, and their design flexibility allows the incorporation of diverse therapeutic agents, including small molecules, peptides, and nucleic acids. Co-delivery capabilities of polymeric systems also offer significant potential for synergistic combination therapies. Indeed, PLGA is one of the most widely utilized biodegradable polymers for controlled peptide delivery systems due to its biocompatibility, tuneable degradation kinetics, and well-established regulatory approval. First synthesized in the 1970s, PLGA undergoes hydrolytic degradation to lactic and glycolic acid metabolites, which are naturally cleared via the Krebs cycle, thereby eliminating long-term toxicity concerns, making it ideal for sustained-release applications [2].

Protein and peptide-based drugs are considered among the most effective therapeutic agents due to their high specificity and strong affinity for biological targets. However, therapeutic peptides face inherent delivery challenges due to their short half-lives (<30 minutes for many), susceptibility to proteolysis, and limited membrane permeability. PLGA-based formulations offer significant advantages for peptide therapeutics, which often suffer from poor bioavailability, rapid clearance, and enzymatic degradation when administered through conventional routes [4]. The ability to encapsulate peptides within PLGA nanoparticles and microparticles enables sustained release over days to months, thereby reducing dosing frequency and improving patient compliance. Nanoparticles (<1 μm) excel at systemic delivery due to enhanced permeation and retention, while microparticles (1 to 100 μm) serve as injectable depots for prolonged action.

Indeed, PLGA microparticles offer several advantages over conventional drug delivery systems. These include: (1) the ability to tailor the rate and duration of drug release by modifying the polymer type and fabrication methods, (2) greater physical and chemical stability compared to other delivery systems such as liposomes, and (3) reduced drug dosing due to enhanced delivery efficiency. In recent years, long-acting injectable (LAI) microspheres have gained significant attention for the delivery of therapeutic proteins and peptides. Their high drug-loading capacity and potential to provide sustained release over extended periods make them particularly suitable for chronic therapies. Key advantages of LAI microspheres include improved drug stability and bioavailability, enhanced therapeutic efficacy, and reduced dosing frequency. More than 20 FDA-approved PLGA formulations have demonstrated clinical viability [2,5].

However, achieving optimal release kinetics requires balancing competing factors: excessive initial burst release risks toxicity, while delayed erosion phases may undermine therapeutic efficacy. The controlled release of peptides from PLGA systems involves complex mechanisms, including diffusion through water-filled pores, polymer matrix erosion, and swelling-induced drug liberation [1,6,7]. Understanding these release mechanisms and their controlling factors is crucial for optimizing therapeutic outcomes and developing clinically viable formulations [2]. This review summarizes current knowledge on peptide release from PLGA nano- and microparticles, examining the fundamental mechanisms, formulation strategies, and factors influencing release kinetics. By integrating fundamental principles with emerging technologies, this work provides a framework for designing next-generation PLGA delivery systems optimized for precision peptide therapeutics.

## Factors controlling peptide release

**Figure 1.**
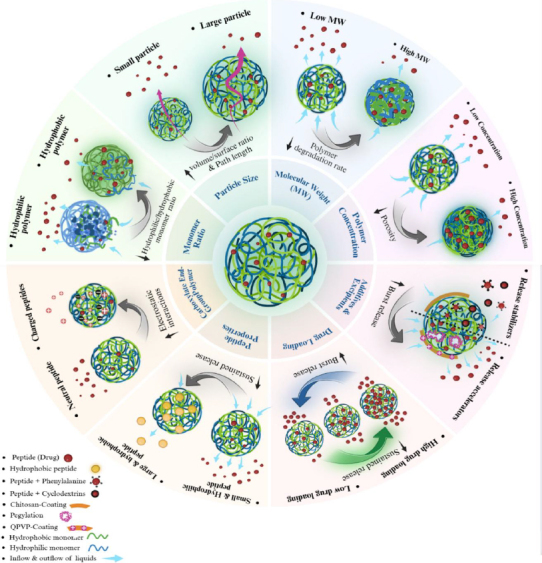
Factors controlling peptide release

In this section, the main factors that influence the peptide release profile from PLGA nano- and microparticles are discussed. These factors include PLGA molecular weight, monomer ratio, particle size, end-group, concentration, peptide properties, drug loading, and additives and excipients. Figure 1 summarizes the role of these factors on peptide release.

### Molecular weight

The molecular weight of PLGA significantly influences peptide release kinetics through its impact on polymer degradation rates and matrix porosity. Lower molecular weight PLGA exhibits faster hydrolytic degradation, leading to increased water uptake and accelerated peptide diffusion [8-10]. In a study of leuprolide acetate release from in situ forming microparticles, PLGA with molecular weight 16,000 Da (RG 502) demonstrated 49.3 % burst release within 24 hours compared to 24.1 % for higher molecular weight PLGA (RG 504, 56,500 Da). Studies have shown that increasing amounts of low-molecular-weight fractions in PLGA lead to higher initial burst release, indicating that both average molecular weight and polydispersity affect release profiles. This phenomenon occurs because low-molecular-weight chains degrade more rapidly, creating preferential pathways for peptide diffusion [10-12].

Conversely, higher molecular weight PLGA extends release duration by slowing degradation and reducing matrix porosity. A neutral peptide required over 8 weeks for complete release from ester-capped 15 kDa PLGA microparticles, compared to 13 days from 7 kDa PLGA. This delayed release results from prolonged polymer entanglement and slower pore formation [13]. In another study by Haim Zada *et al.* [14], Lower MW PLGA (75:25, 30 kDa) NPs released TRH peptide faster than higher MW PLA (55 kDa) NPs, consistent with accelerated degradation of low-MW polymers. Ravivarapu *et al.* [15] evaluated the release of leuprolide acetate from microparticles with various blends of high-MW (28.3 kDa) and low-MW (8.6 kDa) PLGA. As anticipated, blends with higher ratios of high-MW polymer exhibited slower degradation rates and, consequently, longer release periods.

### Monomer ratio (lactide:glycolide)

The lactic acid-to-glycolic acid molar ratio fundamentally determines PLGA degradation rates and, consequently, peptide release [9,16]. Higher glycolic acid content increases polymer hydrophilicity and degradation rate, resulting in faster peptide release [9]. Conversely, higher lactide content slows degradation due to increased hydrophobicity and crystallinity [17]. In a comparative study of anti-angiogenic peptide delivery, 85:15 (lactide:glycolide) PLGA microparticles demonstrated higher encapsulation efficiency and slower degradation compared to 65:35 formulations, supporting prolonged drug release over 6 months [16]. Kranz and Bodmeier also examined the effect of polymer composition (lactide:glycolide ratio) on peptide release. Size-exclusion chromatography revealed that PLGA formulations (RG 502 and RG 503) degraded more rapidly than PLA (R 203), reaching almost complete degradation by day 28, indicating accelerated polymer degradation and peptide release at lower lactide:glycolide ratios [11].

### Particle size

Particle size significantly influences release kinetics by affecting the surface area-to-volume ratio and the diffusion path length [18,19]. Smaller particles generally exhibit faster burst release due to higher surface area, while larger particles provide more sustained release [19,20]. Studies with PLGA microparticles demonstrated that particles smaller than 50 μm showed rapid diffusion-based release, completing within 2 days, whereas larger microparticles (>50 μm) exhibited sustained release patterns continuing for three months [17]. By adjusting particle porosity and size, Santhosh *et al.* [20] achieved controlled release rates ranging from 114.5 to 308.1 ng day^-1^, with more porous and larger particles enabling higher and longer-duration release (up to 42 days). Smaller porous microparticles (1.43 μm average diameter) sustained Neuregulin-1 (Nrg-1) release for up to 21 days, whereas larger particles (2.37 μm average diameter) extended release up to 42 days. The release kinetics exhibited an initial burst phase followed by a sustained release plateau. The larger particles achieved a daily release rate of 251.4 ng day^-1^ *in vitro* and 267 ± 4 ng day^-1^ when suspended in artificial cerebrospinal fluid (aCSF).

For nanoparticles, size effects on release are particularly pronounced. Microfluidic-produced nanoparticles (102 nm) showed increased burst release (70 % after 1 hour) compared to nanoprecipitation-produced particles (189 nm) with 50 % burst release within 1 hour and nearly complete release for both formulations at 24 hours [21]. A study by Han *et al.* [22] demonstrated sustained release of a C5aR1-targeted hexapeptide (HC-[OPdChaWR]) from lipid shell-PLGA core nanoparticles. Nanoparticles fabricated using acetonitrile (ACN) showed rapid release (90.0 ± 9.0 % at 72 h), attributed to a smaller particle size (~150 nm) and lack of lipid shell. The microfluidic method enabled precise control of particle size (30-600 nm) and PDI (<0.3), which are critical for tuning release kinetics. Larger nanoparticles (~400 nm) produced with DCM showed slower release due to a lower surface area-to-volume ratio. Smaller nanoparticles (<150 nm) from ACN or acetone led to faster diffusion-driven release. Solvent miscibility in water (*e.g.* ACN’s high miscibility) increased nucleation rate, favouring smaller particles and faster release. The prolonged-release profile correlated with improved *in vivo* performance in preclinical models of lung disease.

### End-group and electrostatic interactions

Electrostatic interactions between charged peptides and PLGA degradation products critically influence release kinetics [13,23]. As PLGA degrades, it generates negatively charged carboxylic acid groups that strongly bind positively charged peptides, significantly impeding their diffusion. Balmert *et al.* [13] showed that peptides with higher net positive charge released significantly slower: a peptide with +3.1 charge *per* kDa exhibited <20 % cumulative release over 58 days, compared to near-complete release of a neutral peptide within 13 to 58 days, depending on PLGA type. Maximum daily release rates also declined with increasing charge, from ~6.9 % day^-1^ for neutral peptides to <1 % *per* day for highly cationic peptides. This charge effect was consistent across PLGA types, including 7, 15 and 43 kDa. In leuprolide delivery systems, PLGAs with free carboxylic acid end groups exhibited lower initial release (7.2 %) compared to ester-terminated variants (32.0 %), attributed to electrostatic interactions between leuprolide's basic groups and the acidic polymer terminus [8].

The acidic microenvironment created during PLGA degradation (pH as low as 2.2) increases peptide protonation and binding [12,24]. This effect is particularly problematic for cationic peptides, where electrostatic interactions can delay release by 15 to 20 days [25]. Strategies to mitigate these interactions include co-encapsulation of basic additives, such as L-histidine, which acts as a proton scavenger and preserves peptide activity [23].

### Peptide properties

Peptide physicochemical properties, including molecular weight, charge, and hydrophobicity, significantly influence release kinetics. Hydrophilic peptides typically show faster release than hydrophobic ones due to weaker interactions with the PLGA matrix [14,26]. In one study, hydrophilic oxytocin exhibited ~50 % burst release within 1 hour and near-complete release within 35 hours, whereas hydrophobic LHRH demonstrated sustained release over weeks due to hydrophobic partitioning [14]. In another study, the hydrophobic 20-mer anti-angiogenic peptide showed prolonged release over 6 months in 85:15 PLGA microparticles [16]. One strategy to mitigate the burst release of hydrophilic peptides is to graft hydrophobic moieties, such as phenylalanine, onto PLGA, or to employ cyclodextrin complexation. These approaches enhance peptide stability within the delivery matrix and effectively reduce the initial burst release [26].

Peptide size also affects diffusion rates, with smaller peptides (<10 kDa) diffusing faster due to reduced steric hindrance. Conversely, peptides exceeding 20 kDa demonstrate significantly slower release kinetics, often requiring polymer erosion for complete liberation [27]. As discussed in section 2.4, higher positive charges correlate with slower release rates due to electrostatic interactions with the PLGA matrix, especially in the case of carboxylic acid-terminated PLGA. One mitigating strategy was to convert peptides to zinc complexes, thereby reducing positive charge and accelerating release by 40 % [25]. Maintaining the secondary structure also influences release behaviour. In a study, cyclic peptides (*e.g.* ciclosporin A) demonstrated slower release (100 days) than linear counterparts due to structural stability [28]. Conformationally sensitive peptides like GDNF require fusion tags (*e.g.* collagen-binding domain) to maintain bioactivity during sustained release, as reported in another study [29].

Additionally, peptide degradation during release remains a significant challenge. Nucleophilic groups, such as N-terminal amines and lysine side chains, can undergo acylation reactions with PLGA ester linkages, forming covalent adducts that hinder drug release. For example, the acylation of octreotide in the commercial formulation Sandostatin LAR^®^ has been shown to influence its release profile [30]. PLGA degradation creates acidic microenvironments, risking peptide denaturation. As mentioned in section 2.4, co-encapsulation of L-Histidine (≥25 wt.%) has been reported to preserve functionality by scavenging protons [23,25]. Peptide aggregation, particularly *via* β-sheet formation, poses another challenge by reducing bioavailability. The use of lyoprotectants such as trehalose has been shown to prevent aggregation during storage [27].

### Drug loading

Drug loading, the mass ratio of peptide to polymer, fundamentally influences release profiles by modulating matrix porosity and diffusion pathways. Higher loading usually increases burst release due to elevated concentration gradients and greater surface-associated peptide but can also accelerate sustained release by creating interconnected pores [31,32]. TP508-loaded PLGA/PEG microparticles exhibited 0.23 to 0.29 mg mg^-1^ release at 5 wt.% loading versus 0.44 to 0.51 mg mg^-1^ at 15 wt.% loading due to higher surface peptide density [33]. Conversely, plectasin-loaded PLGA nanoparticles showed 100 % burst release at 0.625 wt.% loading versus 77 % at 2.5 wt.% loading, as higher loading shifted peptide distribution toward the core [34]. PLGA microparticles loaded with >30 wt.% protein developed continuous pore networks, shifting release from degradation-controlled to diffusion-dominated kinetics. High-loading vancomycin nanoparticles (90 wt.%) released 58 % of the drug within 24 hours due to interconnected aqueous channels [31].

These findings indicate that drug loading values can be optimized to prevent excessive burst release and ensure controlled rates in the sustained-release phase. While too low and too high drug loadings can accelerate burst and sustained release, respectively, an optimal loading can concentrate most of the peptide cargo at the particle core to prevent burst release while hindering excessively interconnected network formation, which can trigger accelerated sustained release.

### Polymer concentration

In PLGA formulations, it critically modulates peptide release by influencing matrix density, pore structure, and degradation kinetics. Higher PLGA concentrations generally reduce burst release and extend sustained-release duration by forming denser matrices with limited pore interconnectivity, whereas lower concentrations accelerate release through increased porosity and faster solvent diffusion [31]. This effect must be balanced against reduced total loading capacity. For buserelin acetate ISM, increasing PLGA RG 502 concentration from 20 to 40 % reduced burst release from 49.3 to 25.4 % within 24 hours by limiting solvent diffusion and promoting denser particle formation [11]. Albumin-loaded PLGA microparticles with 10 % polymer concentration released over 14 days, while 5 % concentration formulations completed release in 7 days [35].

### Additives and excipients

Various additives can modulate release profiles in different ways. Medium-chain triglycerides (MCTs) create porous internal structures, shifting from triphasic to continuous biphasic release profiles. In leuprolide microparticles, 20 % MCT increased intermediate-phase release from 25 to 45 % and reduced final-phase release from 57 to 28 % [36]. For Endostar, MCT co-encapsulation improved *in vitro* release from 32.22 % to 79.04 % over 30 days and reduced peptide retention in vivo from 42.25 to 9.87 % [37]. PEG incorporation increases matrix porosity and enhances water uptake, thereby accelerating release. High PEG content (PEG : LA+GA = 1:8) increased TNFR-BP burst release to 39.4 % within 10 hours, compared with 26.7 % for low-PEG systems [32,38]. Co-encapsulation of magnesium carbonate reduces peptide denaturation risk by 40 % in glycolide-rich PLGA, stabilizing pH-sensitive peptides like exenatide [25]. When using PVA as an emulsifier, higher hydrolysis (87 to 89 %) reduces nanoparticle PDI to 0.044, enabling uniform, sustained release of antimicrobial peptides such as Ctn [15-34,39]. Phenylalanine-grafting reduces burst release from 50 to <10 % by enhancing hydrophobic peptide interactions [27]. Lyoprotectants can prevent peptide aggregation during freeze-drying and reduce burst release, maintaining release consistency. In PLGA microparticles, trehalose reduced post-lyophilization size increase by 30 % during storage. Cyclodextrins reduced burst release by 15 to 20 % *via* hydrophobic complexation with peptides [25,40].

Chitosan coating significantly reduces initial burst release by forming a network that restricts water infiltration and peptide diffusion. In recombinant human interleukin-2) rhIL-2(delivery systems, chitosan coating reduced burst release from 70.18 to 44.85 % [41-43]. Polycationic coatings, such as quaternized poly(4-vinylpyridine) (QPVP), alter release dynamics by enhancing water penetration and modifying surface charge. While coated nanoparticles showed lower initial burst (4 *vs.* 9 % for uncoated), they demonstrated sustained release profiles with enhanced degradation [44]. Factors affecting peptide release from PLGA nanoparticles and microparticles are shown in Table 1.

**Table 1.** Factors influencing peptide release from PLGA nano and microparticles

Factor	Impact on release	Mechanism	Optimization strategy
PLGA MW	Higher MW correlates with slower release	Delayed degradation rate at higher MW	Commercial PLGA products with various MWs are available - Mix Multiple MW ranges for particle synthesis to tune release rate
Monomer ratio (lactide:glycolide)	Higher lactide content correlates with slower release	Delayed degradation rate due to higher hydrophobicity	Higher ratios (85:15, 75:25) for chronic conditions and lower ratios (50:50, 65:35) for acute conditions
Particle size	Smaller size correlates with faster release	Higher surface-to-volume ratios favour surface adsorption rather than core encapsulation	Microparticles suit sustained release applications (chronic) while nanoparticles favour fast release applications (acute)
PLGA carboxylate end-group	Promotes slower release	Electrostatic interactions with basic residues	Co-encapsulation of basic additives
PLGA ester end-group	Generally, promotes faster release than carboxylate PLGA	Lack of electrostatic affinity – Could form covalent adducts with nucleophilic peptide residues and impede release	Co-encapsulation of basic additives
Peptide size	Lower MW (< 10 kDa) correlates with faster release	Reduced steric hindrance	Restricted to therapeutic needs
Peptide charge	Positive charge correlates with slower release	Electrostatic interaction with PLGA-degradation metabolites	Restricted to therapeutic needs
Peptide structure	Hydrophobicity correlates with slower release	Hydrophobic interaction with the PLGA matrix	Adjust monomer ratio to modulate PLGA hydrophobicity. When rapid hydrophilicity-mediated is problematic: modify with hydrophobic moiety, cyclodextrin complexation, or chitosan coating
Drug loading	Higher drug loading correlates with faster sustained release – Low drug loading risks premature release	High: Formation of interconnected pore network – Low: Surface adsorption	Must be optimized to balance burst release and accelerated sustained release. Can use chitosan coating to prevent burst release
PLGA concentration	Higher concentration correlates with slower release	Denser matrix and limited interconnectivity	Must be balanced to prevent limited drug loading – Can use PEG and MCT to enhance interconnectivity

## Release mechanisms from PLGA systems

PLGA-based peptide delivery systems typically exhibit a triphasic release profile, comprising an initial burst, a lag phase, and a sustained release phase [2,18]. This pattern reflects the complex interplay of diffusion and degradation mechanisms operating at different stages of peptide release [17,31].

Phase I: An initial burst of release occurs within the first 24 to 48 hours and accounts for 10 to 45 % of total peptide release [25]. This phase primarily results from surface-associated or weakly bound peptides that readily diffuse into the release medium [18,31]. The magnitude of burst release primarily depends on particle size, peptide loading, and surface morphology [19,45].

Phase II: Lag phase represents a period of slow, diffusion-controlled release lasting days to weeks [25,31]. During this phase, peptide diffusion through the intact polymer matrix is limited, resulting in minimal peptide release [18].

Phase III: Erosion-driven release begins as polymer degradation accelerates, creating pores and channels that facilitate sustained peptide release [31]. This phase typically exhibits zero-order kinetics and can last for weeks to months, depending on polymer properties [2,18].

Smaller PLGA particles or formulations with rapid degradation often exhibit simplified biphasic release patterns, consisting of an initial burst followed by sustained release without a distinct lag phase. This occurs when particle degradation proceeds faster than the time scale required for complete drug depletion [31,46].

## Formulation strategies for controlled release

### Single oil-in-water emulsion and double water-in-oil-in-water emulsion

Emulsion-based methods are among the most widely used techniques for the synthesis of nanoparticles and microparticles, as they are compatible with a broad range of drugs with diverse solubility profiles. The single-emulsion technique (oil-in-water, O/W) is particularly well suited to encapsulating hydrophobic drugs. In this approach, PLGA and the drug are first dissolved in a small volume of a volatile organic solvent. This organic phase is then added dropwise to an aqueous phase containing a stabilizer, typically polyvinyl alcohol (PVA). The resulting mixture is homogenized by sonication and stirred for a defined period to ensure complete evaporation of the organic solvent, thereby forming stable particles. The double emulsion method is employed when the active agent to be encapsulated is hydrophilic. This technique is commonly used for the encapsulation of proteins, peptides, and various hydrophilic drugs. In this method, the active compound is first dissolved in an aqueous phase, which is then added to a PLGA solution in an organic solvent, forming a primary water-in-oil (W/O) emulsion. This primary emulsion is subsequently added to a second aqueous phase containing a stabilizer, and the mixture is homogenized to form a water-in-oil-in-water (W/O/W) emulsion. The organic solvent is then evaporated under stirring, resulting in the formation of hardened particles. The product is collected by centrifugation or ultrafiltration and washed to remove unreacted components. Finally, the particles are dried, allowing them to remain stable for prolonged periods [7,25,47].

### Spray drying

This approach offers scalable, one-step production with precise control over particle properties. Particles are formed by atomization of the emulsion through a hot air stream and fast solvent evaporation. Poor solvents, such as methanol, promote early PLGA shell formation but increase the risk of surface drug migration, whereas fast-evaporating solvents, such as dichloromethane, limit burst release to ~15 %. Different atomization techniques, including two-fluid, three-fluid, and ultrasonic atomization, have limitations in scalability, size control, and peptide destabilization, respectively [25,48].

### Inkjet printing

The inkjet printing technique is another scalable approach, owing to large-scale nozzle arrays, and provides exceptional control over particle size and morphology. This method has achieved 54 and 93 % encapsulation efficiency for octreotide and ciclosporin A, respectively, with sustained release over 49 to 100 days. Limited residual solvent in the final product is another advantage of this technique. Several parameters of the involved materials and printing equipment influence particle properties, and the optimization process can be complex [28].

### Precipitation

This simple and cost-effective method yields particles with moderate encapsulation efficiency. Particles are formed by the gradual addition of an organic water-miscible solvent containing the polymer into an aqueous phase. Non-aqueous nanoprecipitation has achieved 100 % encapsulation efficiency for neuropeptides while minimizing premature drug leakage [14,49].

### Flash nanoprecipitation

Compared to conventional nanoprecipitation, flash nanoprecipitation (FNP) employs turbulent mixing in confined mixing vessels within milliseconds to yield highly uniform particles. This advanced technique enables high loading (up to 90 wt.%) and encapsulation efficiency (>90 %) while maintaining particle stability. Moreover, FNP minimizes peptide exposure to denaturing interfaces [31].

### Microfluidic methods

Using a microchannel-equipped chip, this highly reproducible approach produces pico-scale emulsion droplets to provide precise control over particle size and polydispersity, resulting in more consistent release kinetics [17,21,22]. Lipid-shell PLGA core nanoparticles produced via microfluidics demonstrated prolonged release with 37 % cumulative release at 72 hours compared to 90 % for conventional methods [22].

### Supercritical fluid

**Figure 2.**
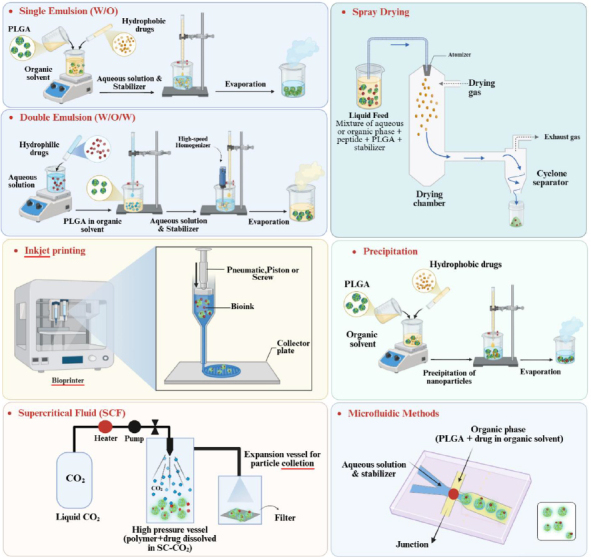
Formulation strategies for controlled release

In this relatively newer method, particles are formed by rapid depressurization of a saturated polymer solution by nozzle transfer. CO_2_ has been a popular candidate as a saturating agent due to enabling mild temperature and pressure conditions and eliminating organic solvents from the process. Key parameters to control in this technique include temperature, pressure, polymer concentration, and nozzle diameter [17,50]. Table 2 summarizes the advantages and disadvantages of each formulation strategy and Figure 2 provides a graphical summary of each strategy.

**Table 2.** Advantages and disadvantages of peptide-loaded PLGA particle formulation methods

Formulation method	Advantages	Disadvantages
Single emulsion (O/W) and double emulsion (W/O/W)	enables encapsulating both hydrophilic and hydrophobic peptides; well-established protocols have enabled its application in many commercial products; enables particle synthesis in both nano and micro scale; adaptable to batch and continuous modes	Surfactants complicate the purification process; numerous influencing parameters complicate reproducibility; oil/water interface compromises peptide stability; substantial burst release
Spray drying	Scale up possibility – Precise control over particle size – Simple one-step protocol	The two-fluid, three-fluid and ultrasonic atomization approaches exchange trade-offs on scalability, control on particle size, and peptide stability
Inkjet printing	Precise control over particle size and morphology; scalability; high encapsulation efficiency; limited solvent residue	High encapsulation efficiency for hydrophilic peptides require pre-cooling the anti-solvent; high internal porosity; numerous influencing parameters from material and equipment require optimization
Precipitation	High encapsulation efficiency- especially for hydrophilic peptides; reduces burst release; limits peptide stability issues	Multiple residual solvents to remove complicate the purification process and raise taxological concern; scale-up challenges; batch-to-batch variability
Microfluidic methods	Precise control over particle size and morphology; reduced batch-to-batch variability; high encapsulation efficiency; limits peptide stability issues	Scale-up challenges; process complexity and parameter sensitivity; incompetence for producing microparticles; quality control challenges
Supercritical fluid	Environmentally friendly and solvent-free; Excellent process control and reduced batch-to-batch variability; High encapsulation efficiency	Limited PLGA and hydrophilic peptide solubility in supercritical CO_2_; expensive equipment; challenges in controlling aggregation and particle size; scale-up challenges

## Advanced delivery systems

PLGA micro- and nanoparticles have garnered significant attention as efficient drug delivery systems due to their ability to improve the pharmacokinetics and therapeutic index of encapsulated drugs. These carriers provide controlled and sustained drug release, reduce drug clearance, and enhance bioavailability, making them ideal for both systemic and localized delivery. PLGA protects sensitive drugs from enzymatic and chemical degradation, enabling the delivery of a broad spectrum of therapeutics, including anticancer agents, anti-inflammatory drugs, and antibiotics. Moreover, the versatility in formulation methods allows the encapsulation of drugs with diverse physicochemical properties, addressing solubility and stability challenges commonly encountered in conventional drug delivery.

### Multi-compartment systems

Nano-in-micro systems: These systems combine the advantages of both size scales, with nanoparticles providing cellular uptake and microparticles offering sustained release. PLGA nanoparticles encapsulated within yeast cell wall particles demonstrated reduced burst release and prolonged hypoglycemic effects of exenatide peptide, exceeding 12 hours [51].

Core-shell architectures: This approach enables sequential release profiles and improved stability. Lipid-shell PLGA core nanoparticles showed 33 % reduction in burst release compared to PLGA-only formulations while maintaining prolonged antihypertensive effects of KY5 peptide for 144 hours [52].

### Stimuli-responsive systems

pH-responsive systems: These systems exploit physiological pH variations for targeted delivery. Enteric-coated PLGA nanoparticles showed limited gastric release of VLPVPR peptide (<40 % at pH 1.0) while enabling complete intestinal release (100 % at pH 7.4) within 8 hours [53].

Enzyme-responsive systems: These systems use specific enzymatic activity to enable controlled release. PLGA/collagen microparticles containing collagen-binding GDNF exhibited enzyme-dependent release rates ranging from 8 to 250 ng mg^-1^, depending on collagenase concentration [29].

## *In vitro-in vivo* correlations

**Figure 3.**
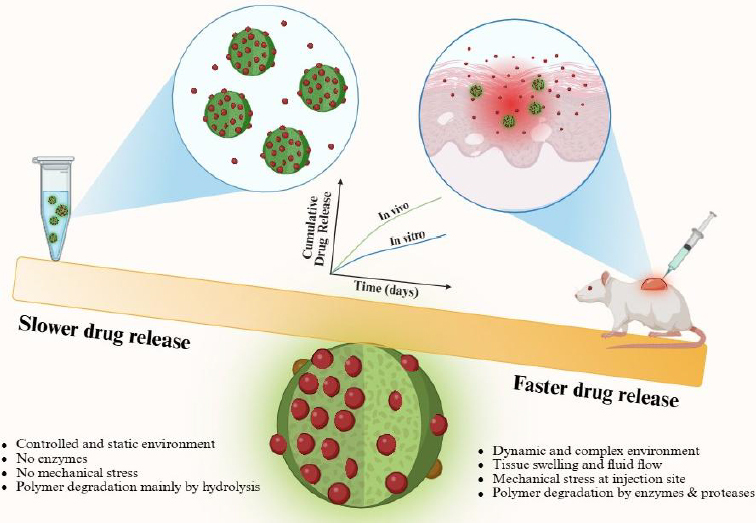
The gap between *in vitro* and *in vivo* release assay results

Establishing predictive relationships between *in vitro* and *in vivo* release remains challenging for PLGA-peptide systems, as indicated in Figure 3. Peptide release often occurs more rapidly *in vivo* than *in vitro* due to enhanced swelling, enzymatic degradation, and mechanical stress at injection sites. Comparative studies reveal significant variations in *in vivo*/*in vitro* ratios across different peptides and formulations. For example, exenatide formulated with 28 kDa PLGA was entirely released over 63 days *in vitro* but only 28 days in vivo, with *in vivo*/*in vitro* ratios of 1.72, 1.85, and 1.08 at 10, 50 and 100 % release, respectively. Strategies to improve correlation include using release media that mimic in vivo environments, incorporating serum proteins, and developing formulation-specific models [53,54].

## Clinical applications and marketed products

Several PLGA-based peptide delivery systems have achieved clinical success, demonstrating the technology's commercial viability. Lupron Depot^®^ utilizes 75:25 PLGA microspheres containing 10 % leuprolide acetate for monthly testosterone suppression in prostate cancer treatment. Suprecur^®^ MP, Decapeptyl^®^ and Trelstar™ Depot are other marketed PLGA-based formulations for prostate cancer [55,56]. Nutropin Depot^®^ is a marketed product for pediatric growth hormone deficiency, which contains growth hormone-loaded PLGA microparticles [57]. Sandostatin LAR^®^ Depot (Octreotide acetate) and Somatuline^®^ LA (Lanreotide) have reached the market for acromegaly therapy [58]. Bydureon^®^ represents the first FDA-approved PLGA formulation for GLP-1 receptor agonists, encapsulating exenatide in ~60 μm microparticles for sustained release over 7 weeks [59]. These commercial successes validate the use of PLGA-based microcarriers for peptide delivery in the treatment of various diseases.

Table 3 summarizes the information on these marketed products. As shown in Table 3, many of these products employ a range of lactide:glycolide ratios to modulate release kinetics according to the application. Lower ratios are used when rapid onset and burst release are favoured, while higher ratios are preferred for sustained release and reduced dosing frequency.

**Table 3.** Marketed PLGA-based products for peptide delivery

Product name	Active Ingredient	Formulation	Manufacturing technique	FDA approval date	Disease
**Lupron Depot^®^**	Leuprolide acetate	PLGA (75:25) microparticle (10-30 μm)	Double emulsion	1989	Prostate cancer
**Suprecur^®^ MP**	Buserelin acetate	PLGA (50:50) microparticle (< 200 μm)	Spray drying	Not approved by FDA	Prostate cancer
**Decapeptyl^®^**	Triptorelin pamoate	PLGA (75:25, 85:15, 90:10, 95:5) microparticle (10 to 110 μm)	Double emulsion, solvent-free microgranule process, premix membrane emulsification	Not approved by FDA	Prostate cancer
**Trelstar™ depot**	Triptorelin pamoate	PLGA (74:26, 77:23, 52:48) microparticle (< 250 μm)	Solvent-free microgranule process	2000	Prostate cancer
**Nutropin Depot^®^**	Somatropin	PLGA (50:50) microparticle	ProLease cryogenic process	1999	Pediatric growth hormone deficiency
**Sandostatin LAR^®^ depot**	Octreotide acetate	PLGA (55:45) microparticle (30 to 100 μm)	Precipitation	1998	Acromegaly
**Somatuline^®^ LA**	Lanreotide	PLGA (75:25, 50:50) microparticle (20 to 100 μm)	Double emulsion	2007	Acromegaly
**Bydureon^®^**	Exenatide	PLGA (50:50) microparticle (median: 55 μm)	Precipitation	2012	Diabetes

Currently, no marketed products based on PLGA nanoparticles for peptide delivery are available. Manufacturing complications and regulatory issues have prevented PLGA nanoparticles from entering the pharmaceutical peptide market. There is a lack of robust manufacturing methods for PLGA nanoparticles at the industrial scale, as well as limitations in analytical methods for characterization and quality control. An absence of established regulatory pathways for PLGA nanomedicines further complicates marketing of PLGA nanoparticle-based peptide drugs [60]. Marketed PLGA-based products for peptide delivery are shown in Table 3.

## Conclusion

PLGA nanoparticles and microparticles represent highly versatile and clinically validated platforms for delivering therapeutic peptides, which are often characterized by poor stability, rapid degradation, and short biological half-lives. These biodegradable carriers offer tenable and sustained release profiles through the careful selection and manipulation of polymer composition (*e.g.* lactic/glycolic acid ratio and molecular weight), surface properties, particle size, and formulation techniques such as single- or double-emulsion, nanoprecipitation, or spray-drying. By adjusting these parameters, it is possible to modulate release kinetics to match therapeutic needs, ranging from rapid initial release to long-term depot formulations that last days or even weeks. A deep understanding of the interplay between diffusion, polymer erosion, and swelling enables the rational design of delivery systems that ensure controlled release while minimizing peptide denaturation or aggregation. Moreover, PLGA particles can protect sensitive peptides from enzymatic degradation in the gastrointestinal tract or in the systemic circulation, thereby enhancing their bioavailability and efficacy. Despite these advantages, challenges persist, including incomplete release, unpredictable *in vitro - in vivo* correlation, and the risk of peptide acylation during formulation. However, advances in polymer chemistry, peptide stabilization strategies (*e.g.* PEGylation, pH modulation), and scalable manufacturing techniques are steadily overcoming these limitations. The clinical success of PLGA-based peptide formulations, such as Lupron Depot^®^ and Sandostatin LAR^®^, demonstrates the viability of this technology in achieving effective, patient-friendly, and commercially successful long-acting release systems. These achievements provide a robust foundation for the development of next-generation PLGA-based platforms tailored to emerging peptide therapeutics, particularly in oncology, endocrinology, and metabolic disorders.
